# Individual Differences in Inhibitory Control, Not Non-Verbal Number Acuity, Correlate with Mathematics Achievement

**DOI:** 10.1371/journal.pone.0067374

**Published:** 2013-06-13

**Authors:** Camilla Gilmore, Nina Attridge, Sarah Clayton, Lucy Cragg, Samantha Johnson, Neil Marlow, Victoria Simms, Matthew Inglis

**Affiliations:** 1 Mathematics Education Centre, Loughborough University, United Kingdom; 2 School of Psychology, University of Nottingham, United Kingdom; 3 Department of Health Sciences, University of Leicester, United Kingdom; 4 Institute for Women’s Health, University College London, United Kingdom; Cardiff University, United Kingdom

## Abstract

Given the well-documented failings in mathematics education in many Western societies, there has been an increased interest in understanding the cognitive underpinnings of mathematical achievement. Recent research has proposed the existence of an Approximate Number System (ANS) which allows individuals to represent and manipulate non-verbal numerical information. Evidence has shown that performance on a measure of the ANS (a dot comparison task) is related to mathematics achievement, which has led researchers to suggest that the ANS plays a critical role in mathematics learning. Here we show that, rather than being driven by the nature of underlying numerical representations, this relationship may in fact be an artefact of the inhibitory control demands of some trials of the dot comparison task. This suggests that recent work basing mathematics assessments and interventions around dot comparison tasks may be inappropriate.

## Introduction

We live in an increasingly numerically-oriented society. Every day when we shop, travel or communicate we are required to make decisions based on quantitative information. Is there enough fuel in the car to reach my destination? Which telephone payment plan is better for me? How much is a sale item with a 10% discount? Individuals’ success in dealing with numbers and quantities is related to their job prospects, income and quality of life [1]. Despite the critical importance of numerical skills, not only in our daily life but also to ensure a skilled workforce, many Western societies are failing in attempts to improve the numerical skills of young people [2,3]. As a result, researchers have increased their efforts to understand the cognitive bases for mathematical skill with a view to developing more effective teaching strategies.

Much work has focused on understanding how numerical and quantity information is represented. Recently psychologists have proposed the existence of an Approximate Number System (ANS) which supports the representation and processing of nonsymbolic numerical quantities. Studies have shown that adults, children and infants are able to use this system to compare, order and add sets of items presented as arrays of dots or sequences of tones [4–7]. These findings have led researchers to propose that the ANS may serve an important role in the learning of mathematics [8].

A number of studies provide evidence in support of a link between the ANS and mathematics learning. Specifically, children’s performance on nonsymbolic numerical tasks has been found to correlate with their scores on standardized or curriculum measures of mathematics [8–14]. On the basis of these findings, some researchers have developed mathematical interventions or assessments which incorporate nonsymbolic measures [15] (or www.panamath.org). However, not all studies have found links between nonsymbolic number performance and mathematics achievement in children [16–21] and the evidence for a relationship in adult participants is mixed [22–27].

**Figure 1:**
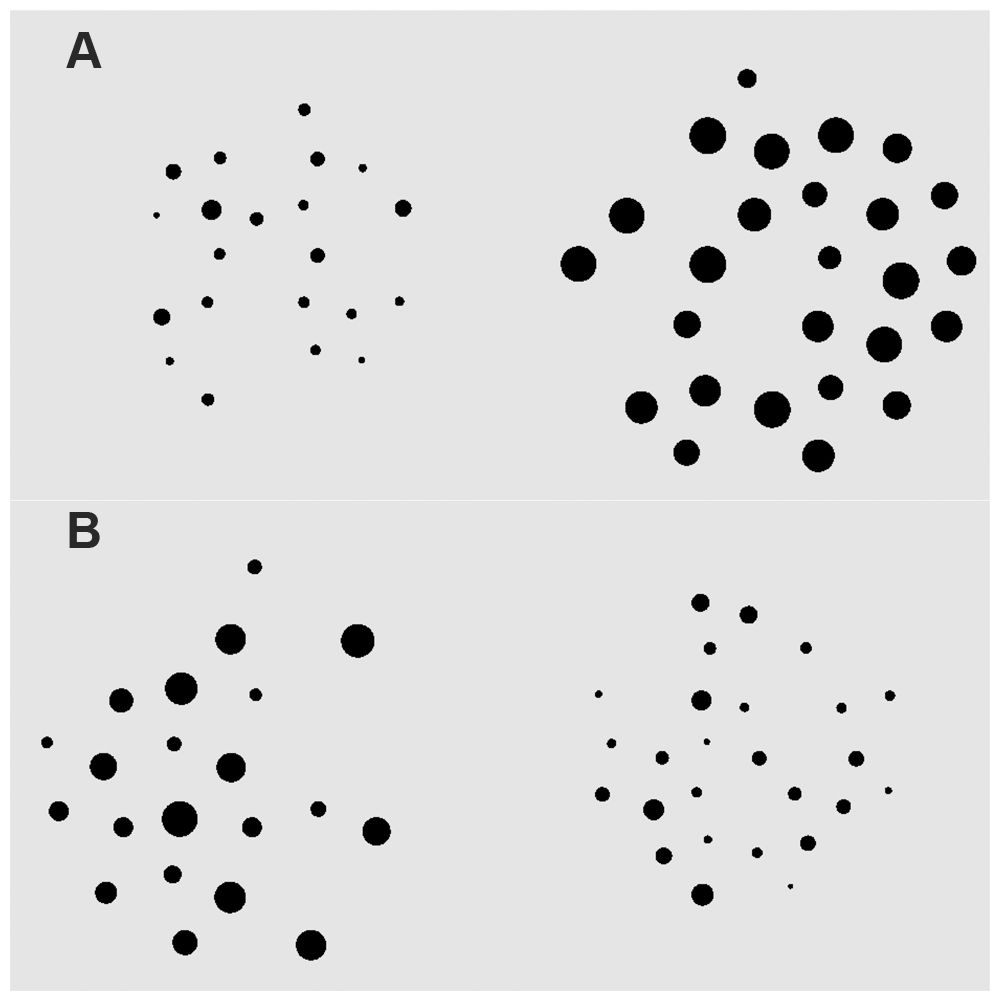
Congruent and incongruent dot comparison task trials. Both pairs of images show the trial 21 vs. 26 dots but (A) is a congruent trial, where the more numerous array has larger dots and a larger area, and (B) is an incongruent trial, where the more numerous array has smaller dots and a smaller area.

Studies that investigate the relationship between nonsymbolic number processing and mathematics achievement have typically used a dot comparison task to measure nonsymbolic number performance. In these tasks participants are shown two arrays of dots and are asked to select the more numerous array while disregarding other features of the images, such as the dot size and arrangement. To ensure that participants cannot use superficial characteristics of the dots to choose the more numerous array, researchers have attempted to control for continuous quantity variables such as dot size, density and total area. The most commonly-used method for doing so is to produce different sets of images in which these variables are either positively or negatively correlated with number across a pair of arrays [28,29]. For example, on some trials participants may be shown two arrays in which the more numerous array has larger dots and a larger area (*congruent* trial, see Figure 1A), while on other trials the more numerous array has smaller dots and a smaller area (*incongruent* trial, see Figure 1B). In this way researchers have demonstrated that children and adults can select the more numerous of two arrays with above chance accuracy, and that performance on this task does not result simply from responding to the continuous quantity features of the arrays.

A consequence of creating the dot array images in this way is that on congruent trials of the dot comparison task the visual characteristics of the arrays provide an additional cue to number, but on incongruent trials participants need to inhibit a response based on these salient characteristics and respond only to the number of dots. As a result, the incongruent trials of the dot comparison task bear strong resemblance to a Stroop task [30]. In a typical Stroop task participants are shown color words written in different-colored inks. Their job is to name the ink color while inhibiting the salient color name. In the same way, when solving incongruent trials of a dot comparison task, participants must first inhibit a response based on the salient visual characteristics of the array and respond instead on the basis of number. The Stroop task is commonly-used as a measure of inhibitory control and therefore it is likely that performance on incongruent trials of the dot comparison task will reflect not only the precision of participants’ numerical representations, but also their inhibition skills. This leads to an intriguing possibility. We know that children’s inhibitory control skills are strongly related to their symbolic mathematics achievement [31–34]. It is therefore possible that the relationship between dot comparison tasks and mathematics achievement, rather than resulting from the precision of underlying numerical representations, is instead driven by the inhibitory control demands of some trials of the dot comparison task.

This hypothesis is yet to be tested. In a previous study [8] Halberda and colleagues found that performance on a dot comparison task was associated with mathematics achievement. Although they included a large number of covariates, including executive function skills, in their exploration of the links between dot comparison performance and mathematics achievement, their study had an unusual retrospective correlational design. They found that children’s 3^rd^ Grade (age 8-9) performance on a standardized mathematics test was correlated with performance on a dot comparison task measured in 9^th^ Grade (age 14-15) after controlling for executive function skills measured in 3^rd^ Grade. Executive function skills were measured using the Contingency Naming Test (CNT), in which children are required to switch between naming an item’s shape or color depending on characteristics of the stimuli. Given that executive function skills develop throughout childhood at different rates [35,36], the retrospective design of this study makes drawing conclusions problematic. It is possible that if children’s inhibition skills are measured at the same age as completing the dot comparison task, rather than 6 years earlier, they might account for the relationship with concurrent mathematics achievement.

Some evidence in support of this proposal was provided by Fuhs and McNeil [37]. They explored the link between dot comparison performance and mathematics achievement in a group of preschool children from low-income backgrounds. They found that performance on the nonsymbolic comparison task was a borderline predictor of scores on a standardized mathematics test for this group of children, but that it was no longer a borderline predictor once inhibition scores were taken into account. They suggested that inhibitory control might be an important mediator of the link between nonsymbolic performance and mathematics achievement in preschool children, and particularly those from a low-income background.

The hypothesis that inhibitory control skills account for the association between performance on a dot comparison task and mathematics achievement leads to two clear predictions. First, there should be a different relationship between dot comparison performance and mathematics achievement for incongruent and congruent dot comparison trials. Specifically, we would expect a significant, positive correlation with mathematics achievement only for the incongruent trials which require inhibitory control. Second, we would expect that performance on a dot comparison task should no longer act as a significant predictor of mathematics achievement once inhibition skills have been accounted for. Here we present two experiments that test these predictions. In the first experiment we test the relationship between mathematics achievement and congruent vs. incongruent dot comparison trials in children aged 4-11 years, and in the second experiment we explore the relationship between performance on a dot comparison test, inhibition skill and mathematics achievement in children aged 8-10 years.

## Experiment 1

## Method

Participants. The participants were 80 children (46 male) who attended the University of Nottingham’s Summer Scientist Week (www.summerscientist.org) – a research and outreach event in which children visited the University with their families and took part in research studies as well as activities and games. Children’s ages ranged from 4.7 to 11.9 years (M=7.7, SD=1.9 years). Children came from mid-to high-socioeconomic backgrounds.

Ethics Statement. Parents or carers of all participants provided written informed consent for their child to take part. Due to the age of the participants, children did not sign written consent but all gave verbal agreement prior to testing. The study and consent procedures were approved by the School of Psychology Ethics Committee, University of Nottingham.

Procedure. Children were given two tasks in a single session lasting approximately 20 minutes. First children completed a dot comparison task in which they were shown a red dot array and a blue dot array, presented simultaneously side-by-side on a computer screen. The ratio between the left and right arrays was 0.5, 0.6, 0.7 or 0.8 and the number of dots in each array ranged from 5 to 22. Children were asked to select as quickly and accurately as possible which array was more numerous, and they responded using the left (left array more numerous) or right (right array more numerous) buttons on a five-button response box. As we were primarily interested in congruency effects, the dot arrays were generated following the method of Pica and colleagues [29]. According to this method two sets of images were created in which dot size and envelope area characteristics vary together. In one set (incongruent) the dot size and envelope area were negatively correlated with number of dots, while in the other set (congruent) the dot size and envelope area were positively correlated with number of dots. In each of 128 experimental trials children saw a fixation point on screen for 1000ms followed by the arrays presented for 1500ms and finally a white screen with a black question mark until response. Children could either respond while the dot arrays were on screen or afterwards. Children’s accuracies and mean response times on the whole task and separately for congruent and incongruent trials were used in analyses. For those participants who performed at above chance level on the dot comparison task (n = 57) ANS acuity estimates (w parameters) were also calculated following the method of Inglis and colleagues [22]. The correlation between ANS acuity and accuracy was close to 1, r = -.94, p < .001. However, because ANS acuities cannot be accurately determined for participants who perform close to chance levels, accuracy was used as the primary index of performance.

Following completion of the dot comparison task the calculation subtest of the Woodcock-Johnson III Tests of Achievement [38] was administered. This is an untimed pencil and paper arithmetic test in which participants solve calculation problems of increasing difficulty until six consecutive problems are answered incorrectly. Children’s raw scores on the test were used in analyses.

## Results

Children’s accuracies on the congruent and incongruent trials of the dot comparison task are summarized in Table 1. As expected, children were significantly less accurate on the incongruent trials compared to the congruent trials (*t*(79) = 10.0, *p* < .001). We then explored response times for the different trial types. Correct responses to incongruent trials were significantly slower than correct responses to congruent trials (correct congruent M = 1067ms; correct incongruent M = 1413ms; *t*(79) = 3.54, *p* = .001). Moreover, for incongruent trials correct responses were significantly *slower* than incorrect responses (incorrect incongruent M = 1140ms; *t*(79) = 3.35, *p* = .001), whereas for congruent trials correct responses were significantly *faster* than incorrect responses (incorrect congruent M = 1310ms; *t*(79) = -2.60, *p* = .011). These findings all support the proposal that correctly solving an incongruent trial necessarily requires an additional processing step compared to solving a congruent trial, namely inhibiting a response based on visual characteristics. As a result, differences in RT for correctly-solved incongruent and congruent trials give an indication of participants’ inhibition skills. Children with good inhibition skills would show a smaller difference in RT for incongruent and congruent trials than those with poorer inhibition skills. If, as we propose, inhibition skills underpin the relationship with mathematics achievement then we would expect to find a correlation between the difference in RT for congruent and incongruent trials and mathematics achievement. This was indeed the case. Children with a smaller RT difference between correctly answered incongruent and congruent trials, and thus good inhibition skills, scored higher on the mathematics achievement test than children with a larger RT difference (*r* = -.27, *p* = .015).

**Figure 2:**
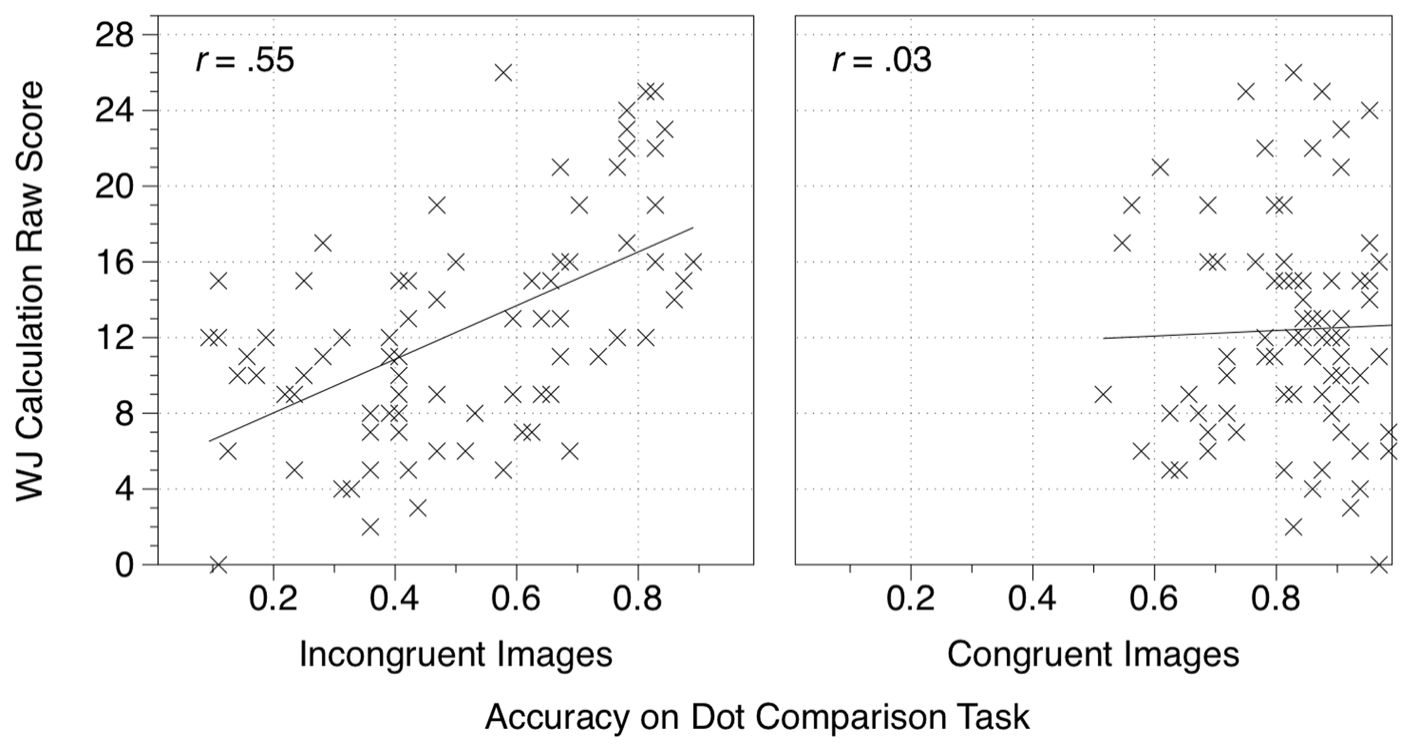
The relationship between calculation scores and dot comparison task performance. In Experiment 1, children’s scores on the Woodcock-Johnson III calculation subtest was significantly correlated with performance on the incongruent, but not the congruent trials of the dot comparison task.

Next to explore whether children’s overall accuracies on the dot comparison task were related to performance on the standardized mathematics test, a series of correlations were conducted. Overall performance on the dot comparison task was significantly related to mathematics achievement (*r* = .57*, p* < .001), however when this relationship was explored separately for the two trial types a different pattern emerged. Performance on the incongruent trials of the dot comparison task was strongly related to mathematics achievement (*r* = .55, *p* < .001) but performance on the congruent trials was not (*r* = .03, *p* = .80). These correlations are significantly different (Williams-Steiger test, *t*(77) = 3.34, *p* = .001; see Figure 2). This pattern of findings was replicated if only the subset of participants for whom *w* estimates could be calculated were included: the correlations for incongruent trials with mathematics (*r* = .55, *p* < .001) and for congruent trials with mathematics (*r* = .03, *p* = .82) were significantly different (*t*(56) = 2.845, *p* = .006).

**Table 1:** Descriptive statistics for measures used in Experiments 1 and 2.

Experiment	Task	Min	Max	Mean	SD
1	Dot comparison congruent trials (accuracy)	.52	1.00	.82	.12
	Dot comparison incongruent trials (accuracy)	.09	.89	.51	.23
	WJ-III calculation (raw score)	0	26	12.41	5.88
2	Dot comparison overall (accuracy)	.60	.93	.78	.06
	WIAT-II numerical operations (raw score)	9	45	21.89	6.68
	NEPSY-II naming (combined score^a^)	3	17	9.70	3.46
	NEPSY-II inhibition (combined score^a^)	4	16	9.74	3.32

a The NEPSY-II combined scores combine accuracy and speed information.

These results show that the relationship between performance on the dot comparison task and mathematics achievement is driven by performance on the incongruent trials. We next considered whether this pattern of results would be replicated in previously-published data involving a different sample of children but similar tasks to the current study. Inglis and colleagues [22] found that there was a significant correlation between scores on a dot comparison task and performance on the Woodcock-Johnson III calculation subtest for children aged 7–9 years but not for adult participants. We re-analysed these data to consider the correlation with mathematics achievement separately for congruent and incongruent trials. Our findings replicated the current results: for children the correlation between mathematics achievement and performance on the incongruent trials of the dot comparison task was significant and positive (*r* = .34, *p* = .04) but the correlation with congruent trials was nonsignificant and negative (*r* = -.22, *p* = .18). These correlations were marginally significantly different (*t*(35) = 1.88, *p* = .068). For the adult participants, who completed a slightly different version of the dot comparison task involving larger numerosities, there was no overall relationship between dot comparison scores and mathematics achievement. However, there was a marginally significant difference in the strength of the correlations between mathematics achievement and either congruent (*r* = -.15, *p* = .15) or incongruent dot comparison trials (*r* = .16, *p* = .11; *t*(97) = 1.75, *p* = .08).

Both sets of results show a similar, clear pattern. The relationship between dot comparison score and performance on a standardized mathematics test is driven entirely by performance on the incongruent trials. This suggests that rather than being driven by precision of underlying representations, this frequently-observed correlation may arise from the domain-general demands of a portion of the dot comparison task. In particular, the incongruent trials, but not the congruent trials, require participants to inhibit a response based on salient features of the dot arrays (such as dot size or area) and to respond solely on the basis of number. Given that there is a wealth of evidence for a strong relationship between inhibition skill and mathematics achievement in children [31–34], it is possible that inhibition skill may thus account for the relationship between dot comparison performance and mathematics achievement. To directly test this hypothesis we conducted a second experiment in which we measured inhibition skill as well as mathematics achievement and performance on a dot comparison task in children aged 8–10 years old.

## Experiment 2

### Method

Participants. The participants were 71 children (36 male) aged between 7.8 and 10.5 years (M=9.4, SD=0.6 years). These children formed the comparison group of typically developing children for a study of prematurity and mathematics skills. Children were individually tested either in a quiet room at their school or at their home. The children were recruited from 67 schools across the Midlands and South East of England and represent a diverse mix of socio-economic status backgrounds.

Ethics Statement. Parents or carers of all participants provided written informed consent for their child to take part. Due to the age of the participants, children did not sign written consent but all gave verbal agreement prior to testing. The study and consent procedures were approved by the NHS, Derbyshire Research Ethics Committee.

Procedure. Children completed a computer-based dot comparison task, the WIAT-II UK Numerical Operations subtest, and the NEPSY-II Inhibition subtest. In addition to these measures, children completed a number of other standardized and experimental tasks that are not reported here. Tasks were split across one or two sessions and children were given breaks between tasks as needed. All sessions were completed within one week for each participant.

In the dot comparison task children were shown two dot arrays (one red, one blue) presented simultaneously side-by-side on a 17″ laptop screen. The ratio between the dot arrays was 0.5, 0.6, 0.7, or 0.8 and the number of dots in each array ranged from 5 to 28. Children were asked to select which array was more numerous as quickly and accurately as possible, and they responded using the A (left array more numerous) or L (right array more numerous) keys on the laptop keyboard. In contrast to Experiment 1, where congruency effects were the focus, in this experiment the dot arrays were generated following the method of Gebuis and Reynvoet [28]. According to this method four sets of images were created: 1) envelope area and dot size are both positively correlated with the number of dots; 2) envelope area is positively correlated and dot size is negatively correlated with the number of dots; 3) envelope area is negatively correlated and dot size is positively correlated with the number of dots; 4) envelope area and dot size are both negatively correlated with the number of dots. This approach does not allow trials to be separated into congruent and incongruent trials in a simple fashion but it does provide a more sophisticated control for continuous quantity variables in cases where overall performance is of primary interest. In each of 80 experimental trials children saw a fixation point on screen for 1000ms followed by the arrays presented for 1500ms and finally a blank screen until response. Children could either respond while the dot arrays were on screen or afterwards. ANS acuity estimates (w parameters) were calculated for all participants and used in analyses.

Children’s mathematical achievement was assessed using the WIAT II-UK Numerical Operations subtest [39]. This is a pencil and paper standardized test in which participants complete a series of increasingly complex calculation problems until six consecutive problems are answered incorrectly. Children’s raw scores on the test were used in analyses. Inhibition skills were assessed using the NEPSY-II Inhibition subtest [40]. In this task children are first shown a series of black and white circles and squares and asked to name them as fast as possible within a time limit. Accuracy and speed on this part of the task are then combined to produce a naming score. Participants are then shown the identical images and this time are required to provide the opposite name for each (i.e. say “circle” for square and “square” for circle), again completing as many as possible within a time limit. Accuracy and speed on this part of the task are then combined to produce the inhibition score, with higher scores indicating better inhibition. Each score integrates the total number of errors made (uncorrected and self-corrected) and total completion time for that condition (maximum 180 seconds for naming and 240 seconds for inhibition). The naming score captures the general demands of the task while the inhibition score captures the specific inhibition demands in addition to these elements.

## Results

We tested whether inhibitory skills could account for the relationship between dot comparison performance and mathematics achievement using a series of hierarchical regression models. With WIAT Numerical Operations raw score as the dependent variable, we first conducted a model in which dot comparison performance (*w* scores) was entered in the first step and inhibition and naming scores from the NEPSY Inhibition subtest were entered in the second step. As shown in Table 2, dot comparison *w* score was a significant predictor of calculation skill when entered in step one and performance on the inhibition task added significantly to the model when entered in step two. A second model was then conducted in which the order of these steps were reversed. This time performance on the inhibition test was a significant predictor when entered in the first step, but dot comparison *w* score did not significantly improve the fit of the model when added in step two. In other words, dot comparison performance did not explain significant variance in mathematics achievement once performance on the inhibition task had been taken into account, but inhibitory control explained significant additional variance over and above that accounted for by dot comparison performance. An identical pattern of results is obtained if accuracy is used in place of *w* estimates. In both models, the inhibition score, rather than the general naming score, from the NEPSY subtest was a significant predictor. This demonstrates that inhibition skills, and not other aspects of the task (e.g. naming speed), accounted for the relationship between scores on the dot comparison task and performance on the mathematics achievement test.

**Table 2:** Linear regression models predicting arithmetic performance from scores on the dot comparison and inhibition tasks in Experiment 2.

Model	Step	Variable	β	ΔR^2^	Sig. ΔR^2^
1	1	Dot comparison *w*	-.35*	.12	.003
	2	Dot comparison *w*	-.16	.16	.001
		NEPSY-II Inhibition: naming score	.14		
		NEPSY-II Inhibition: inhibition score	.37*		
2	1	NEPSY-II Inhibition: naming score	.18	.26	< .001
		NEPSY-II Inhibition: inhibition score	.40**		
	2	NEPSY-II Inhibition: naming score	.14	.02	.172
		NEPSY-II Inhibition: inhibition score	.37*		
		Dot comparison *w*	-.16		

DV = WIAT numerical operations raw score; significance of β weights: *p < .05, **p < .001

## Discussion

Our findings suggest that the commonly-observed relationship between children’s performance on dot comparison tasks and mathematics achievement may be the result of inhibition skills, rather than the precision of nonsymbolic representations. We provide three lines of evidence to support this hypothesis. First, correct responses to incongruent trials on the dot comparison task are slower than both correct responses to congruent trials and incorrect responses to incongruent trials. This indicates that correctly solving incongruent trials requires an additional processing step. Second, the relationship between dot comparison scores and mathematical achievement was significant only for the incongruent trials of the dot comparison task. In these trials, but not the congruent trials, participants must inhibit a response based on the salient visual characteristics of the arrays and respond only on the basis of number. Finally, we demonstrated that performance on a dot comparison task is no longer a significant predictor of mathematics achievement score once inhibition skills have been accounted for.

We suggest that when an individual is faced with a dot comparison trial there are two ways in which they can select a response. One way is to respond simply to the salient visual characteristics of the dot arrays, such as selecting the array with the larger dots, or the array that covers a greater area. On congruent trials of the dot comparison task this approach leads to a correct response and on incongruent trials this leads to an incorrect response. Alternatively, if an individual successfully inhibits a response based on these visual characteristics they will then respond on the basis of ANS representations. This may or may not lead to a correct response, depending on the ratio involved and the precision of the individual’s nonsymbolic representations. As a result, an individuals’ score on a dot comparison task will reflect not only the precision of their nonsymbolic representations, but also their inhibition skills. We propose that it is the inhibition element, rather than the precision of numerical representations element, that results in a significant correlation with mathematics achievement.

Our findings build on previous work which suggested that inhibition skills may be an important component of dot comparison tasks for young children from low income backgrounds [37]. We found that inhibition skills account for the relationship between dot comparison performance and mathematics achievement in school-aged children from a wide variety of backgrounds. In a reanalysis of previously-published data, we even found a marginally significant difference in the strength of the correlation between mathematics achievement and the congruent or incongruent dot comparison trials for adult participants. This suggests that the incongruent trials of the dot comparison task may even challenge the inhibition skills of adults.

It appears to be inhibition skills specifically, rather than executive functions generally or domain-general elements of the task such as speed of processing which account for the relationship between dot comparison performance and mathematics achievement. Halberda and colleagues [8] found that the relationship between dot comparison performance and mathematics achievement remained significant after controlling for performance on the Contingency Naming Test (CNT). However, the retrospective design of that study makes it difficult to draw firm conclusions and the CNT does not provide a specific measure of inhibition skill that is separate from the general demands of the task. In a concurrent correlational study Fuhs and McNeil [37] found that performance on inhibition tasks did reduce the relationship between dot comparison score and mathematics achievement but the authors highlighted that it may have been the more general processing demands of the task, rather than inhibition skills specifically, that accounted for this. Our use of a task which provided both specific inhibition scores, as well as a more general naming score, allows us to demonstrate that it is the inhibition element of the task that is critical.

The dot comparison task in its typical format involves a heavy inhibition element. Alternative versions of a comparison task which lessen this inhibition load are possible. For example Barth and colleagues [41] used a cross-modal comparison task in which participants are asked to select whether a sequence of tones or an array of dots contain more elements. The continuous quantity characteristics of each set are different (i.e. dot size, area, density vs. tone length, frequency) and thus participants are not able to compare the sets on the basis of any of these variables. Kramer and colleagues [42] used displays in which a set of items were represented using second-order visual motion. In these displays visual characteristics such as luminosity, area and density were unrelated to number and therefore could not be used as a cue to numerosity. It has yet to be established whether performance on these types of comparison tasks is related to mathematical achievement but this would be a valuable avenue for research. The present findings suggest that performance on tasks such as these, which do not involve inhibiting salient cues, might be unrelated to mathematics skill.

Our results highlight that there is an important gap in our current knowledge of the ANS and the tasks that are commonly used to measure it. We need to better understand the demands of ANS measures such as the dot comparison task and what factors, in addition to the acuity of numerical representations, contribute to scores on these tasks. More generally, we do not know how the ANS interacts with other domain-general cognitive systems. For example, the role that working memory plays in the representation and processing of nonsymbolic quantities. A focus on examining the links between the ANS and mathematics learning evident over the past few years has meant that important work in better understanding the ANS itself has been neglected.

It remains to be established whether and how the ANS may be involved in the early stages of learning the symbolic number system. However, our findings demonstrate that much existing evidence for a link between individual differences in the ANS and mathematics achievement in children may instead arise from the demands of the task. Efforts to integrate dot comparison tasks into mathematical assessment and intervention [15] (or www.panamath.org) may therefore be inappropriate.
